# Evaluation of Human Osteoblasts on NIPS Micro-Patterned PCL Carriers Containing Nanohydroxyapatite and Reduced Graphene Oxide Using PSµM

**DOI:** 10.3390/molecules27207091

**Published:** 2022-10-20

**Authors:** Burcu Tüzün-Antepli, Şükran Şeker, Ayşe Eser Elçin, Gilson Khang, Yaşar Murat Elçin

**Affiliations:** 1 Tissue Engineering, Biomaterials and Nanobiotechnology Laboratory, Ankara University Faculty of Science, Ankara University Stem Cell Institute, 06100 Ankara, Turkey; 2 Department of BIN Convergence Technology, Department of Polymer Nano Science and Technology, Jeonbuk National University, Jeonju 54896, Korea; 3 Biovalda Health Technologies, Inc., 06830 Ankara, Turkey

**Keywords:** phase separation micromolding, micropatterned cell carrier, osteoblasts, poly(ε-caprolactone), nanohydroxyapatite, reduced graphene oxide, osteogenic differentiation

## Abstract

The content and surface topology of tissue engineering scaffolds are two important parameters in regulating the cell behavior. In this study, a phase separation micromolding (PSµM) method was implemented to develop micro-groove-imprinted poly(ε-caprolactone) (PCL)–nano hydroxyapatite (nHAp)–reduced graphene oxide (rGO) ternary blend constructs. Physical and chemical characterizations of cell-devoid constructs were performed by FTIR, XRD, TGA, DSC, porosity, swelling, wettability analysis, tensile and compression mechanical tests. The in vitro biological performance of human osteoblasts cultured on micro-patterned blend constructs was evaluated by MTT and alamarBlue viability assays. The findings revealed that nHAp and rGO significantly promote cell viability and proliferation, while the micro-pattern determines the direction of cell migration. Alkaline phosphatase and Ca^2+^ analyses were carried out to determine the osteogenic properties of cell-laden constructs. This study describes a simple method to generate topologically modified ternary blend PCL/nHAp/rGO constructs using the PSµM method, which contributes to cell proliferation and migration, which is particularly important in regenerative medicine.

## 1. Introduction

Skeletal injury, cancer, infections, arthritis, neoplasm and failed arthroplasties result in rise of the clinical demand for bone grafts, and in the cases of critical size defects the self-healing of the body is not sufficient. Bone is the second-most transplanted tissue after blood, and bone graft materials are used in millions of clinical operations annually [1,2]. Synthetic bone grafts, also called artificial bone, which stand out with their osteoinductive properties, are increasingly used to repair bone tissue. The individual classes of materials have been unable to fully mimic the properties of bone when used alone. Today, the goal is to combine suitable material properties with bioactivity [3]. Recent studies focus on the development of composite materials based on polymers/bioceramics/natural minerals to mimic the properties of natural bone [3,4].

Poly(ε-caprolactone) (PCL) is a widely preferred polymer especially in bone-tissue engineering research due to its high biocompatibility and relatively slow degradation profile, providing time for osteogenic differentiation, moderate mechanical strength, and ease of handling [5,6,7]. Calcium phosphates, i.e., hydroxyapatite (HAp), and β-tricalcium phosphate (β-TCP), closely resemble the mineral content of natural bone tissue. It has been shown that calcium and phosphate ions exert osteoinductive effects in vivo and HAp can stimulate the endogenous expression of osteogenic growth factors such as bone morphogenetic proteins (BMPs) and increase alkaline phosphatase (ALP) activity in multipotent stromal cells (MSCs) [1,3,8]. Therefore, the osteoconductivity and osteoinductivity of HAp can compensate for the lack of bioactivity of PCL. Recently, graphene-based additives, especially graphene oxide (GO) and reduced graphene oxide (rGO), have attracted attention in biomedical research. With their functional groups and electrostatic properties, graphene-based additives can positively influence interactions with proteins, the microenvironment and also cells, and promote the proliferation and differentiation of progenitor cells [9]. An HAp/rGO nanocomposite has been proposed as a potential scaffold combination to promote new bone formation. The inclusion of rGO has been shown to improve osteogenic differentiation of MC3T3-E1 preosteoblasts without impairing cell viability [10]. The incorporation of rGO into the electrospun PCL scaffold has been shown to increase the attachment, proliferation and neuronal differentiation of adipose stem cells [11]. Therefore, in this study, rGO was included to improve the bioactive property of the scaffold.

Besides molecular composition, the hierarchical organization and structure of materials, especially the surface topology, affect the properties of a cell–scaffold construct. Surface topography influences cell fate through mechanotransduction, in which the cell membrane senses physical stimuli and converts them into intracellular signals [12]. Therefore, several specific cell responses can be induced, such as adhesion, migration, orientation, proliferation, and differentiation [13,14]. The surface topology is especially important for osteoconduction. The osteogenic cells are bound to the scaffold surface through the fibrin clot after implantation. Cells then settle on the surface by retraction of a temporary fibrin matrix. Therefore, binding of fibrin to the scaffold surface is of great importance for cell–scaffold interactions. Surface roughness is another desired feature of cell substrates, as it favors the attachment process [15].

Various methods such as soft lithography, thermal lithography, ion etching, chemical etching, template-based micromolding and direct printing have been developed to produce micro- or nano-patterned polymeric structures [16]. Each of them has some advantages and drawbacks. The optimum production method can be determined by the properties of materials, the targeted pattern size and the intended use [17]. In this study, a phase separation micromolding (PSµM) method was used. Two major advantages of this method are that it makes it possible to shape the polymer film using inexpensive solvent and non-solvent pairs, and produces blends, mixed matrix, composite polymer–nano-filler and polymer–polymer blend materials.

It is noteworthy that the groove, ridge and depth dimensions of the created micropattern vary in scales ranging from millimeters to nanometers. The current limitations of the production methods, the target tissue for the application, the cell type to be cultivated and the materials to be used play important roles in determining the dimensions of the pattern [18]. It has been suggested that smaller channels play a role in cell organization, while larger channels increase perfusion in scaffolds formed layer by layer. One study revealed that MSCs prefer to adhere to flat surfaces rather than grooves when the groove width is greater than 40 µm [19]. Gugutkov et al. showed that both 2D and 3D configurations of aligned nanofibers promote upregulation of osteogenic genes in MSCs, whereas randomly structured nanofibers do not [20]. Mata et al. 2007 [21] suggested that the highest cell proliferation occurs in cylindrical half-grooves with a ridge 10 µm in diameter. Lu et al. 2009 [22] showed that osteoblastic proliferation and osteogenic activity were significantly higher in structures with narrower pattern (14 × 28 × 24 µm) than in constructs with larger channels.

In this study, a micro-groove-imprinted PCL/nHAp/rGO ternary blend construct was developed using the PSµM method. The physical, chemical, mechanical and in vitro biological characterizations of the constructs were carried out. These constructs may have the potential to be used in in vitro cell interactions and/or tissue engineering applications in the future.

## 2. Materials and Methods

**Figure 1:**
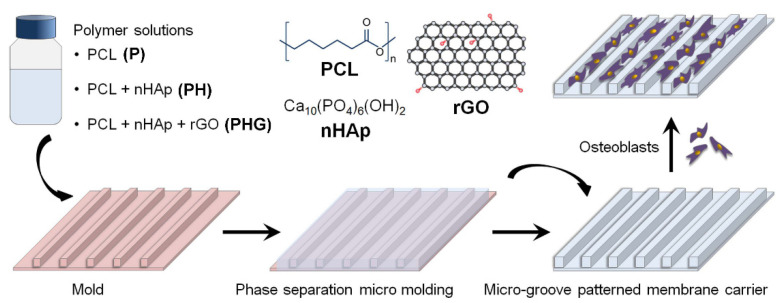
Schematic illustration of micro-patterned polymer/polymer composite membrane formation on silicone mold by the phase separation micromolding (PSµM) method and its use as a human osteoblastic cell carrier substrate.

The experimental steps of the study are presented schematically in Figure 1.

### 2.1. Materials

All the chemicals and solvents used were analytical grade and supplied from Sigma-Aldrich (St. Louis, MO, USA), except otherwise stated. Poly(ε-caprolactone) had a Mw of 80,000 g/mol (1.145 g/mL density, 60 °C melting point). Hydroxyapatite was used in nanopowder form (nHAp; <200 nm; 502.31 g/mol Mw, 1.038 g/mL density). 1,4-Dioxane (100–102 °C boiling point, 1.034 g/mL density) and ethanol (78 °C boiling point, 0.789 g/mL density) were selected as solvent-nonsolvent pairs. Chemically reduced graphene oxide (rGO) was obtained from Grafen Inc. (Ankara, Turkey) with the following properties: 422.69–499.85 m^2^/g BET surface area, 1.91 g/cm^3^ density, 77–87% C/13–22% O composition, and > 600 S/m electrical conductivity. Sterile disposable tissue culture plasticware was purchased from Corning (Corning, NY, USA). Cell culture medium, fetal bovine serum (FBS) and other supplements were provided from Lonza (Basel, Switzerland).

### 2.2. Geometric Dimensions of Micromolds

It is quite important to determine the upper and lower limits of the pattern size and many factors need to be considered, such as the research question to be answered, the scaffold application site, the interacting cell type, the materials used during scaffold fabrication, surface modification, and processing methods. It has been reported that MSCs prefer to attach onto grooves smaller than 40 µm rather than on flat surfaces or grooves larger than 40 µm [19]. In another study, it was reported that 8 µm-wide microgrooves strongly affect osteoblast attachment, but microgrooves larger than 20 µm have a weak effect on osteoblasts [22]. In addition, there are other studies that point to the suitability of narrow channels for control alignment of osteoblasts [21,23]. Guided by the previous studies mentioned above, substrate micropatterns with dimensions (10 µm and 20 µm) predicted to have a greater impact on cell migration and morphology were created.

In the present study, monocrystalline silicon was first patterned by lithography and deep reactive ion etching; these techniques offer size options even at the nanoscale. The possible limitation in the current study was due to both the nature of the PCL and the casting method (non-solvent-induced phase inversion). If the targeted pattern size is chosen too small, the pattern cannot be transferred onto the polymer and the pattern may be swallowed due to shrinkage. In the light of this information, it was decided to use 10 and 20 µm as the pattern dimensions. Pattern dimensions decided for the production of silicon wafers are given in Table 1.

### 2.3. Preparation and Optimization of Micropatterned Carriers

**Figure 2:**
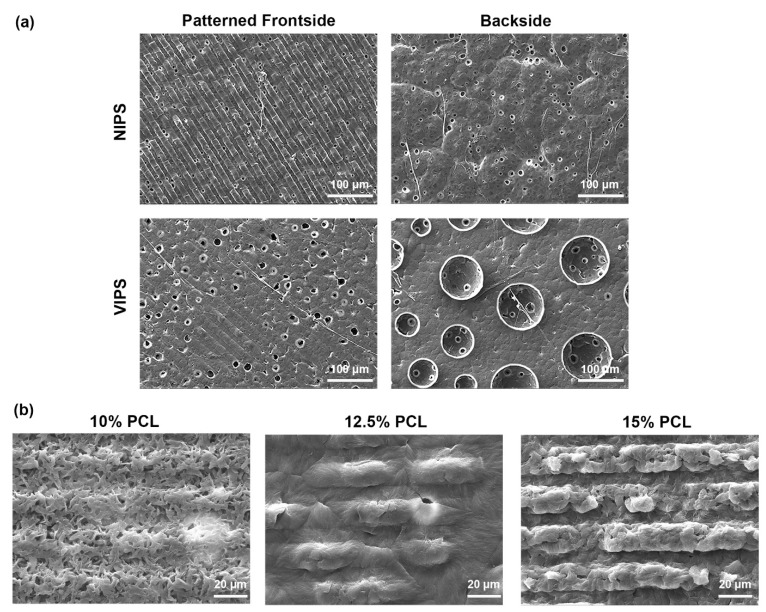
Preliminary SEM evaluations. (**a**) Morphological comparison of PCL membranes produced by NIPS and VIPS methods via SEM. While pattern formation occurs on the frontside of NIPS-applied membranes, this is not the case for those produced with VIPS. Note the 5–10 µm-sized holes on the backside of the NIPS membrane, and the 50–100 µm-diameter craters on the backside of the VIPS membrane. (**b**) Comparison of pattern formation levels when using different PCL concentrations.

Phase separation can be initiated in different ways. At the beginning of this study, the suitability of two methods, the non-solvent-induced phase separation (NIPS) and vapour-induced phase separation (VIPS) methods, were searched for the PCL–nHAp–rGO ternary blend system. In NIPS, the polymeric film is exposed to liquid non-solvent, whereas in VIPS, the polymeric film is exposed to vapor of the non-solvent. Although the composition of the polymeric film was the same in both methods, the morphology of the resulting membranes was different from each other (Figure 2a). In VIPS, pattern formation was not continuous at the polymer film–mold interface due to the large spongy pores. Patterns could be created intermittently. Crater-type holes were observed at the polymer film–solvent interface. On the other hand, NIPS provided complete mold formation and therefore NIPS was chosen as the main fabrication method in this study (Figure 2a). Briefly, in the NIPS method, the ingredients were dispersed homogeneously in 1,4-dioxane solvent. The thermodynamically stable polymeric solution was spread onto the silicon mold and immediately immersed into an EtOH nonsolvent bath [24]. The thermodynamic stability was disturbed by touching with non-solvent EtOH, which initiated phase inversion. By diffusion of solvent into the non-solvent bath and sorption of the non-solvent by polymer, the polymeric solution was separated into two phases, which are called polymer-rich and polymer-lean phases. Later on, the polymer-rich phase forms polymer precipitate and the polymer-lean phase forms pores. Then, the precipitated polymeric film was peeled off from silicon mold. The reverse replica of the mold was printed on the surface of the film. The expected pattern properties of the polymeric carrier would be as given in Table 2. 

The chemical compositions of the carriers produced using the solvent (10 mL 1,4-dioxane) and non-solvent (200 mL EtOH) pair are presented in Table 3. The amount and ratio of the components and the solvent–non-solvent pair were determined based on previous trial-and-error experiments. The amount of solvent was selected as 10 mL, because this volume was sufficient to spread across the whole surface of the silicon mold, which is approximately a disk with a 4-inch diameter. Initially, it was planned to evaluate three different concentrations of polymer: 10%, 12.5% and 15%. The concentrations of the polymeric solutions were calculated according to Equation (1).

(1) $$\%\;polymer\;concentration=\frac{wt.\;of\;polymer}{wt.\;of\;polymer+wt.\;of\;solvent}\times 100$$

It was found that the polymer solution at 10% PCL was not suitable for performing the process, since its low viscosity resulted in slipping out of the mold during spreading. 12.5% PCL concentration was at the appropriate viscosity so that the solution could spread homogeneously and be kept within the boundaries of the mold. However, preliminary SEM analysis revealed that homogeneous and complete pattern formation could not be obtained at this concentration, and the pattern could be formed intermittently (Figure 2b). Although the 15% PCL concentration showed some resistance due to its high viscosity, it could be spread homogeneously along the mold’s surface, resulting in a continuous and homogeneous pattern formation. As the concentration of the polymer solution increases, the higher viscosity leads to a more stable solution, reducing the phase inversion rate by creating resistance for solvent–non-solvent diffusion. The prolonged phase separation provides time for setting patterns on the polymer surface.

Due to the known osteoinductive property of HAp, it was aimed to use HAp in the highest possible amount without preventing the proper pattern formation in the polymer composite. The optimal amount of HAp was found to be 20% by weight of the dry mass of PCL. rGO was a component of one of the composites evaluated. Due to its potential toxicity, rGO was used in accordance with the literature in terms of safety [9]. Table 3 depicts the chemical compositions of the polymeric solutions used for the solvent (10 mL 1,4-dioxane) and non-solvent (200 mL EtOH) pair. In the following sections, the created constructs will be mentioned with the codes given in Table 3.

### 2.4. Morphological, Chemical and Structural Characterizations

#### 2.4.1. Analysis of the Surface Morphology of Constructs via SEM

The patterned surface of the polymeric carrier was cut into circular disks about 1 cm in diameter. Then, the constructs were passed through a graded ethanol series (50–95%) to remove the water. Before analysis, samples were coated with a thin layer of Au/Pd to provide electrical conductivity. The samples were scanned at 20 kV voltage with 30–40 nm resolution using an Evo 40 model SEM device (Zeiss, Jena, Germany) at different magnifications.

#### 2.4.2. ATR-FTIR Analysis

The chemical compositions of P, PH and PHG constructs were investigated by Attenuated total reflectance-Fourier transform infrared (ATR-FTIR) spectroscopy using a Perkin Elmer 400 model device (Waltham, MA, USA) in the spectral range of 3400–400 cm^−1^ with 4 cm^−1^ resolution averaging 16 scans.

#### 2.4.3. XRD Analysis

The phase composition and crystallinity of the constructs were examined by X-ray diffraction (XRD) analysis using an Ultima-IV model X-ray diffractometer (Rigaku, Austin, TX, USA) equipped with Cu Kα radiation (at 40 kV and 20 mA) and a Sol-X energy dispersive detector. Film samples were cut into squares of 1 cm × 1 cm and scanned between 2θ = 5° to 2θ = 60° with 0.02 degree steps and a 2θ/min scanning rate.

### 2.5. Thermal and Physical Characterizations

#### 2.5.1. DSC Analysis

A Perkin Elmer Diamond model (Shelton, CT, USA) differential scanning calorimeter was used to determine the melting temperature (T_m_), melting enthalpy (ΔH_m_), and degree of crystallinity X_c_ (%) of the constructs. For this analysis, at least 20 mg of sample was heated from room temperature to 130 °C with a heating rate of 10 °C/min, then cooled to 0 °C and held at this temperature for 10 min before being heated again to 100 °C.

#### 2.5.2. TGA Analysis

The thermal stability of the constructs was determined by thermogravimetric analysis (TGA) using the Pyris 1 model instrument (Perkin Elmer Inc., Waltham, MA, USA) between room temperature and 600 °C, at a heating rate of 5 °C and 1 µg resolution in a nitrogen atmosphere. At least 20 mg of sample was used in each analysis.

#### 2.5.3. Contact Angle Analysis

The wettability (i.e., hydrophilicity) of the constructs was determined using the Attension Theta Flex model (Biolin Scientific, Gothenburg, Sweden) contact angle and surface tension measuring device. Water contact angle measurements were carried out according to the sessile drop method. Construct samples of 1.5 × 1.5 cm were attached to the flat substrate and 2 µL of deionized water was dropped onto the film surfaces; we then waited for 5 s. At least five measurements were taken per sample.

#### 2.5.4. Porosity Analysis

The percentage porosity of the constructs was estimated based on the ratio of the difference between bulk volume and skeletal volume to bulk volume as indicated in Equation (2).

(2) $$Porosity\;\left(\%\right)=\frac{bulk\;volume\;\left({\mathrm{cm}}^{3}\right)-skeletal\;volume\;\left({\mathrm{cm}}^{3}\right)}{bulk\;volume\;\left({\mathrm{cm}}^{3}\right)}\times 100$$

where:

bulk volume (cm^3^) = thickness (cm) × length (cm) × width (cm)
skeletal volume (cm^3^) = dry weight (g)/density (g/cm^3^)

The samples were dried in an oven at 37 °C for at least one night before weighing. The density of the neat PCL construct (P) was taken as 1.145 g/cm^3^ (neat PCL density). The estimated densities of PH and PHG composite constructs were calculated based on the ratios of density and amount of the components they contain (Table 3).

#### 2.5.5. Swelling Analysis

The constructs were cut into small square pieces, in 1.5 × 1.5 cm size. The dry masses of the samples (W_dry_) were determined by weighing after they were kept in an oven at 37 °C for 24 h. Equilibrium swelling masses of the constructs (W_wet_) were recorded by keeping the samples in phosphate-buffered saline (PBS) at 37 °C for 24 h to reach the equilibrium swelling state. The swelling ratio (%) was calculated according to Equation (3).

(3) $$Swelling\;ratio\;\left(\%\right)=\frac{\left(Wwet-Wdry\right)}{Wdry}\times 100$$

### 2.6. Mechanical Characterizations

#### 2.6.1. Tensile Test

The samples were cut into rectangular shape (10 mm × 30 mm) in which the long side of the sample corresponded to the direction of the pattern. The samples were pulled in the same direction as the pattern orientation until rupture by using a Shimadzu Autograph AGS-X model instrument (Kyoto, Japan) with a 500 N load cell at a crosshead speed of 5 mm/min under atmospheric conditions.

#### 2.6.2. Compression Test

To perform a reliable compression test, a three-dimensional (3D) sample form was created from the membrane constructs [24]. First, the samples were cut into small square pieces of 1 cm × 1 cm. Then, a cube of ~1 cm^3^ (1 cm × 1 cm × 1 cm) was obtained by superimposing 20 pieces and gluing the corners of the pieces with a few drops of 10% PCL polymer solution in dimethylformamide (DMF). Each film was overlapped on the other so that the pattern orientation of each new layer coincided with the pattern orientation of the underlying layer at a 90° angle. A compression test was performed using a Shimadzu Autograph AGS-X device with 500 N load at 1 mm/min speed under atmospheric conditions. The test was stopped when the construct was compressed to 80% of its initial thickness (~0.2 cm).

### 2.7. In Vitro Biological Characterizations

#### 2.7.1. Indirect In Vitro Cytotoxicity Analysis

The potential toxicity of the ingredients and residual solvent and nonsolvent in the constructs were analyzed by the indirect in vitro assay according to ISO 10993-5 standard [25]. Human osteoblasts (hOBs) obtained from ATCC (hFOB 1.19; ATCC^®^ CRL11372™, Manassas, VA, USA) were cultured in DMEM-F12 (1:1) supplemented with 10% FBS, 2 mM L-glutamine, and 100 µL/mL penicillin–streptomycin (Pen/strep) (Standard Medium; SM) in an incubator with settings of 37 °C, 5% CO_2_, 95% air, and >95% humidity until they reached 80% confluence. Cells were then harvested with 0.25% trypsin/EDTA solution and seeded in 24-well plates at a density of 5.0 × 10^4^ cells/well. On the other side, the constructs were sterilized by keeping them in 70% EtOH solution for 2 h, then washed three times with sterile PBS to remove any remaining EtOH. Sterilized constructs were transferred to 24-well culture plates and kept under culture conditions for 24 h to obtain the Extraction Medium (EM). After 24 h, the EM was used to incubate the hOBs for 48 h [26]. An MTT [(3-[4,5-dimethylthiazol-2-yl]-2,5-diphenyl tetrazolium bromide)] assay (Sigma) was used to determine the viability of hOBs cultured in EM. After 48 h of incubation, EM was removed and cells were rinsed with sterile PBS. First, 270 µL of serum-free DMEM-F12, followed by 30 µL of MTT reagent (5 mg/mL) was added to each well.

During 4 h incubation at 37 °C, purple coloured formazan crystals were formed due to mitochondrial dehydrogenase activity of the living cells. The medium was then removed and 400 µL of 0.1 N HCl in anhydrous isopropanol solution was added to each sample to dissolve the formazan crystals. The absorbance [A] values were measured at 570 nm using a SpectraMax M5 model microplate reader (Molecular Devices, Sunnyvale, CA, USA). Relative cell viability was calculated based on Equation (4).

(4) $$Relative\;cell\;viability\;\;\left(\%\right)=\frac{{\left[A\right]}_{s}}{{\left[A\right]}_{NC}}\times 100$$

where [*A*]_S_ is the absorbance value of the tested sample, and [*A*]*_NC_* is the absorbance of the negative control. For comparison, cells cultured in fresh SM were used as negative control, whereas cells treated with 200 mM H_2_O_2_ were used as positive control.

#### 2.7.2. In Vitro Hemocompatibility Analysis

An in vitro hemocompatibility analysis was performed according to ISO 10993-4 standard [27]. Prior to testing, samples (~10 mg in weight) were washed with PBS and sterilized under UV light (254 nm) for 40 min. Anticoagulated blood was diluted 1:20 by mixing with PBS. Each sample was dipped in 2 mL diluted blood in a sterile centrifuge tube. In this experiment, fresh blood diluted with PBS at 1:20 ratio was taken as negative control and fresh blood diluted with distilled water at a ratio of 1:20 was taken as positive control. Afterwards, samples and controls were incubated at 37 °C for 1 h, and then centrifuged at 1500 rpm for 10 min, and the absorbances of the supernatants were measured at 545 nm using a microplate reader (Molecular Devices). The percentage of hemolysis was calculated according to Equation (5).

(5) $$Hemolysis\;\left(\%\right)=\frac{(\left[A{]}_{Sample}-{\left[A\right]}_{NC}\right)\;}{(\left[A{]}_{PC}-{\left[A\right]}_{NC}\right)}\times 100$$

where [*A*]*_Sample_* is the absorbance value of the tested sample, [*A*]*_NC_* is the absorbance of the negative control, and A*_PC_* is the absorbance of the positive control.

#### 2.7.3. Seeding and Culturing of hOBs on the Micro-Patterned Constructs

Micro-patterned films were first cut into ~0.98 cm diameter circular constructs. For sterilization, they were first exposed to UV light (254 nm wavelength) for 30 min and then kept in 70% EtOH solution for 3 h. Finally, residual EtOH in the samples was rinsed in sterile PBS four times for 30 min. hOBs were cultured in DMEM-F12 (1:1) medium supplemented with 10% FBS, 2 mM L-glutamine, and 100 µL/mL Pen/strep (SM) in an incubator set to 37 °C, 5% CO_2_, 95% air, and >95% humidity until they reached 80% confluence. Fresh medium changes were made every other day using routine procedures [28]. When the desired cell density was reached, the hOBs were harvested using 0.25% trypsin–EDTA. Cells were seeded on sterilized constructs at a density of 300,000 cells per construct. The culture of cell-laden constructs was maintained in 24-well plates in SM in the CO_2_ incubator for 14 days.

##### MTT Assay

The cell viability of hOBs cultured on the micro-patterned constructs was also investigated using the MTT test. For that, cell-laden constructs were transferred to new 24-well plates at specified time points (1, 7, and 14 days), and analyzed as described in Section 2.7.1. As a control group, hOBs were seeded on tissue culture plastic at an initial density equivalent to that applied to the membranes, but separated from the plastic as cell plaques within 1-3 days (possibly due to limited surface area and contact inhibition). On the other hand, P-1 and P-2 can be considered positive controls for the PH and PHG groups.

##### alamarBlue Assay

The cell viability of hOBs cultured on the micro-patterned constructs was analyzed using the alamarBlue^®^ assay (Thermo Fisher Scientific, Waltham, MA, USA). At specified time points (1, 3, 5, 7, 10 and 14 days), cell-laden constructs were transferred to new 24-well plates; the medium and serum residues were rinsed with sterile PBS. One milliliter of alamarBlue^®^ dye (1/10) was added into the construct to completely cover the surface. The constructs were incubated inside the CO_2_ incubator for 150 min. They were then removed, and the fluorescence intensities (excitation = 560 nm and emission = 590 nm) of the solutions remaining in the wells were measured with the microplate reader. Cell-free constructs were used as negative control. As mentioned in MTT Assay Section, the positive control (hOBs seeded on tissue culture plastic at a cell density equivalent to that of membranes) could not be sustained due to detachment of cells from the plates within 1–3 days.

#### 2.7.4. SEM Analysis of Cell-Laden Constructs

SEM analysis was performed to evaluate the interaction between cells and the surfaces of the micropatterned constructs, as well as the proliferation, migration and extracellular matrix deposition of cells on the constructs. At specified time points, cell-laden constructs were removed from culture and fixed in 2.5% glutaraldehyde solution overnight. The constructs were then rinsed three times with PBS and dehydrated by passing through graded EtOH series (50–95%). The samples were attached to the stubs, sputter-coated with a thin layer of Au/Pd to provide electrical conductivity, and scanned with the Evo 40 model SEM instrument (Zeiss, Jena, Germany) at 20 kV.

#### 2.7.5. Calcium and ALP Analyses

The osteogenic potential of the cell-laden constructs was characterized by alkaline phosphatase activity and calcium deposition. First, cell-loaded constructs were prepared as described in Section 2.7.4, and cultured in SM supplemented with dexamethasone (10^−8^ M), ascorbic acid (50 µg/mL), and β-glycerophosphate (10 mM). At 10, 20 and 30 days of culture, the culture media were removed for ALP and Ca^2+^ analysis. Extracellular ALP levels were analyzed using the Quantichrom ALP assay kit (Bioassay Systems, Hayward, CA, USA). Extracellular Ca^2+^ levels were analyzed by using the Quantichrom calcium assay kit (Bioassay Systems).

#### 2.7.6. von Kossa and Alizarin Red S Stainings

Extracellular calcium deposition (mineralization) on cell-laden membranes was followed by von Kossa and Alizarin red S histochemical stainings. At predetermined time points (days 0, 10, 20, and 30), constructs were removed from culture, washed with PBS, and kept overnight in 2.5% glutaraldehyde solution for fixation. Prior to analysis, samples were washed with dH_2_O to remove excess glutaraldehyde. For von Kossa staining, samples were kept in 5% (*w*/*v*) silver nitrate solution (Sigma Aldrich) for 1 h. The reaction was terminated by exposure to 5% sodium thiosulfate solution for 5 min. Before photographing, the samples were washed with dH_2_O and dried at room temperature. For Alizarin red S staining, constructs were immersed in 2% Alizarin red-S solution (*w*/*v*; pH 4.2) (Sigma Aldrich) for 5 min. Then, they were removed, washed with 70% EtOH solution, dried at room temperature and photographed.

### 2.8. Statistical Analysis

All experiments were carried in triplicate and the results are depicted as mean ± standard deviation. Statistical analysis was performed by using the ANOVA test in GraphPad Prism 7 (GraphPad Software, La Jolla, CA, USA). Significance levels were considered as * *p* ≤ 0.05, ** *p* ≤ 0.01, *** *p* ≤ 0.001, **** *p* ≤ 0.0001.

## 3. Results and Discussion

### 3.1. Surface Morphology Analysis of Neat Constructs via SEM 

SEM micrographs of cell-free micropatterned P, PH, and PHG constructs are collectively presented in Supplementary Figure S1. rGO and nano-sized HAp could not be observed by SEM analysis as they were embedded in the polymer matrix. It was found that the construct content did not have a significant effect on the pattern dimensions. The polymer surface was obtained as a reverse copy of the silicone mold. That is, the channel of the groove in the mold is processed as the ridge region of the groove in the polymer, and the region of the ridge projection of the groove in the mold is processed as the channel region of the groove on the polymer membrane’s surface. The measured channel and ridge widths of the P, PH and PHG surfaces created in two different micropatterns (Patterns 1 and 2) using the data obtained from the ImageJ program are presented in Table 4. It was determined that the dimensions of the patterns created on the membranes were quite similar to those in the mold, with a difference of ~1–2 μm. A small reduction in membrane pattern size during pattern transfer from the mold has also been reported in other studies [18]. Possible reasons for this may be due to the nature of the polymer and the phase separation method, as well as the pattern sizes selected for the mold. In addition, the porosity formed on the polymer surface may have caused the dimensions to change by creating tortuosity in the patterns.

### 3.2. FTIR Analysis

**Figure 3:**
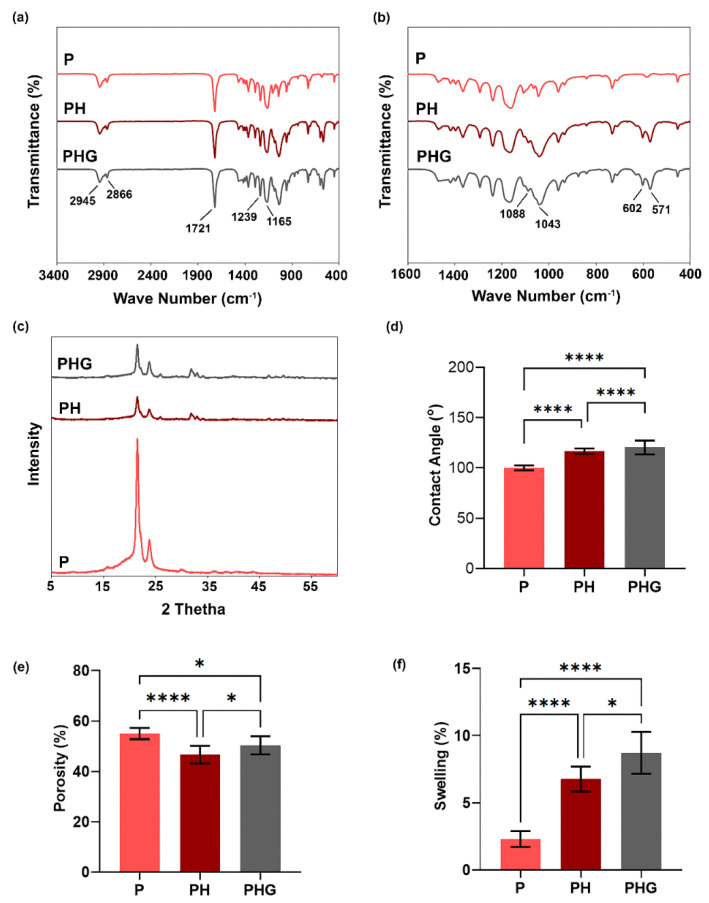
Physicochemical characterization of P, PH and PHG membrane constructs. FT-IR analysis in the spectral range of (**a**) 3400–400 cm^−1^, and (**b**) 1600–400 cm^−1^. (**c**) Crystal structure analysis. (**d**) Wettability, (**e**) porosity, and (**f**) swelling properties. Statistical significance; * *p* ≤ 0.05, **** *p* ≤ 0.0001.

The results of the FTIR-ATR analysis of P, PH, and PHG in the wavenumber band of 400-3600 cm^−1^ are presented in Figure 3a,b. The observed absorption at 2945 cm^−1^ is related to the asymmetric stretching of the C–H hydroxyl group, while the one at 2866 cm^−1^ is associated with the symmetrical stretching of the C–H hydroxyl group. Besides, the absorption at 1721 cm^−1^ is attributed to the stretching vibration of the −C=O bond of the ester carbonyl group. Symmetrical stretch signals of the C–O–C group were observed at 1165 cm^−1^, and asymmetric ones at 1239 cm^−1^. These absorption bands are specific fingerprints of PCL [29] demonstrating that the production method was not effective on the functional groups of PCL. The absorption wavenumbers of the functional groups of the HAp material can be observed with a closer look, within the range of 400–1800 cm^−1^. Two noticeable, sharp peaks associated with the stress and buckling frequencies of the ${\mathrm{PO}}_{4}^{-3}$ (ν4) group of HAp are present at 571 cm^−1^ and 602 cm^−1^. The (ν3) type vibration of the ${\mathrm{PO}}_{4}^{-3}$ functional group was observed at 1088 cm^−1^ and 1043 cm^−1^ [30].

In the infrared spectrum of the rGO-containing constructs, the absorptions of the OH stretching vibration in the -OH functional group, the asymmetrical CH_2_ tension, the symmetrical CH_2_ stretching, and the fractional vibrational absorption of the aromatic C=C group were expected to be observed at 3446 cm^−1^, 2925 cm^−1^, 2860 cm^−1^ and 1627 cm^−1^, respectively [31]. Similarly, the absorption of the C=O group was expected to occur at 1650 cm^−1^, whereas that of the C–OH and C–O groups were expected at 1190 cm^−1^ and 1000 cm^−1^, respectively [32]. Presence of peaks at 1583 cm^−1^, 1735 cm^−1^, 1069 cm^−1^, 1233 cm^−1^ corresponding to the C=C, C=O, C–O and C–OH stretching modes, respectively, have also been reported for rGO [33]. However, the FTIR spectrum of rGO-containing PHG was observed similar to that of PH, with the expected characteristic peaks for rGO not observed. This can be attributed to the very low content of rGO compared to pure PCL and HAp and therefore to the weaker absorption intensity it can create.

Apart from the characteristic peaks in P, PH and PHG, which are considered to be the fingerprints of the parent material, a peak at 875 cm^−1^ was detected in PHG. This wave number is not a characteristic peak of PCL, HAp and rGO, but can be considered to be the result of the combined action of these three materials in PHG.

### 3.3. XRD Analysis

Figure 3c shows the XRD patterns of P, PH and PHG constructs. In sample P, sharp peaks detected at 2θ = 21.486°, 2θ = 22.091° and 2θ = 23.807° are associated with (110), (111) and (200) plane positions, respectively, and are indicative of the crystal structure of PCL. On the other hand, a humped flat curve compared to the baseline between 2θ = 15° and 2θ = 25° degrees indicates the amorphous structure of PCL. These findings confirmed that PCL has a semi-crystalline structure [34] and the production method did not alter this structure. 

In sample PH, distinctive peak formations were observed, some of which were associated with PCL and some specific for HAp. An XRD analysis with reference material, hydroxyapatite (ICDD 9-432) [35] confirmed the presence of HAp in the PH composition. This was interpreted as the formation of a harmonious structure by adding HAp to PCL, which looked like a single material. 

The XRD pattern of PHG (Figure 3c) was very similar to that of PH, and no specific peak was observed for rGO. Similar results have been obtained in other studies of composites in which rGO was incorporated in small amounts as an additive [36]. One possible explanation could be the use of rGO in a very small amount (0.0458% of the biomaterial) in this study. This result may also be due to the amorphous structure of rGO.

Detailed XRD peak analysis demonstrates that there are no peaks at 2θ = 12.93°, 22.126°, 22.91°, 29.93°, 30.31°, 42.04° angles for P and PH. Additionally, it is known that rGO does not form very distinct and sharp peaks due to its amorphous structure [32]. A weak flat peak was observed at 2θ = 42.5°, again with a hump-like graph that starts at 2θ = 12.5° and fades out at 2θ = 35° and reaches its local maximum at ~ 2θ = 27°. The characteristic XRD peaks of commercial rGO have been reported at 2θ = 23.76° and 2θ = 42.74° [37]. In this study, the mounds detected at 22.126°, 22.91°, 29.93° and 30.31° were considered to be a shadow of this hump graph of rGO and the weak mound detected at 42.04° was also considered to be a fingerprint of rGO.

### 3.4. Contact Angle Analysis

The results of the contact angle analysis performed with micropatterned surfaces (pattern #1: C: 20 µm, R: 10 µm, D: 20 µm) are summarized in Figure 3d. Water contact angles for P, PH and PHG were measured as 100.10° ± 2.37°, 116.50° ± 2.69°, 120.38° ± 6.90°, respectively. As a usual consequence of the hydrophobic nature of PCL, the contact angles of the samples were determined to be between 100–120° in accordance with the literature [38].

Although an increase in the wettability of the composite could be expected by adding hydrophilic HAp to the hydrophobic PCL, this was not observed, contrary to the literature [38,39]. It is thought that HAp nanoparticles could not create the expected effect because they were trapped in the polymer bulk structure. Another factor that increases hydrophobicity is considered to be micropatterns [40]. It can be interpreted that the pinning effect of the micropattern increases the water contact angle by creating a second energy barrier for the diffusion of water. rGO also has hydrophilic properties [41], but the amount contained in the PHG construct was too low to make an impact on the contact angle.

### 3.5. Porosity Analysis

The change in porosity with respect to contents of the constructs is depicted in Figure 3e. While the porosity of P was estimated to be 55%, this value decreased to 47% with the addition of HAp to the polymer matrix. The observed decrease in construct porosity caused by HAp addition is consistent with similar studies in the literature [42]. Basile et al. (2015) [43] claimed that nanocrystalline HAp particles form a stronger bond with the polymer chain, resulting in a denser chain packing. Aerts et al. 2000 [44] stated that fillers and additives can increase the viscosity of a polymer solution. High viscosity reduces the mixing rate of solvent–non-solvent during phase inversion. In general, slow coagulation and delayed phase inversion suppress pore formation. The incorporation of rGO into the PH slightly increased the porosity of the construct (from 47% to 50%). This finding is consistent with some studies revealing that the incorporation of rGO into the matrix increases the porosity of the construct [45]. Sánchez-González et al. (2018) [46] reported, as a result of SEM analysis, that the addition of rGO to PCL decreases the surface porosity while increasing the porosity and pore size in the cross-sectional area.

### 3.6. Swelling Analysis

Swelling rates were found to be 2.3%, 6.8% and 8.7% for P, PH and PHG, respectively (Figure 3f). The increase in the swelling rate of constructs with HAp addition is consistent with the studies in the literature [47] and is attributed to the hydrophilic property of HAp [43,48]. The observed increase in swelling as a result of adding rGO to PH is consistent with the small increase in water contact angle and wettability. The observations are supported by the evidence suggested by Seyedsalehi et al. (2020) [49]. The fact that the pinning effect observed in the contact angle analysis performed in 5 s and the findings of the 24 h water swelling test do not appear to be compatible can be attributed to the difference in the surface and volume properties of the materials. 

### 3.7. DSC Analysis

**Figure 4:**
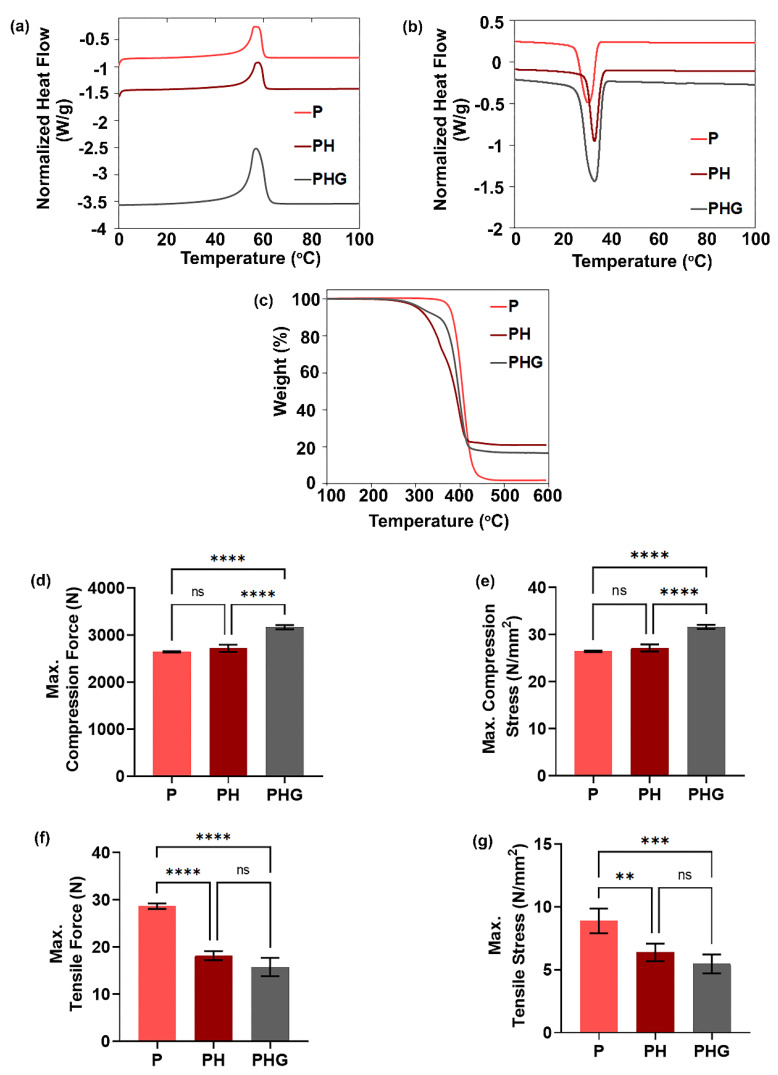
Thermal evaluation and mechanical characterization of the constructs. DSC analysis: (**a**) second heating, and (**b**) cooling. (**c**) TGA thermograms. (**d**) Maximum compressive force, and (**e**) maximum compressive stress at 80% compression. (**f**) Maximum tensile force, and (**g**) maximum tensile stress at rupture. Statistical significance; **p ≤ 0.01, ***p ≤ 0.001, ****p ≤ 0.0001, ns: not significant.

The secondary heating and cooling thermograms of the DSC analysis are presented in Figure 4a,b. The addition of HAp and rGO to the polymer caused a significant shift in the temperature curve. Crystallization temperature (T_c_) and melting temperature (T_m_) increased partially with the addition of HAp and rGO. Compared with P, the increase in T_c_ is ~9%, and the increase in T_m_ is ~3% for PH. In addition, decreases in crystallization and melting enthalpies were observed as ~23% and 18%, respectively (Supplementary Table S1). These findings are in agreement with other studies in the literature [43]. A partial increase in critical temperatures was evaluated as an indicator of compatibility between polymer and additives [50]. The decrease in melting and crystallization enthalpies was attributed to the heterogeneous nucleation points of HAp, a ceramic and mineral origin additive [51]. With the inclusion of HAp in the polymer content, the total crystallinity of the material decreased from 41% to 37%, consistent with the results of Kim and Koh (2013) [52].

The addition of rGO to PCL/HAp did not cause any change in T_c_ and T_m_ values compared to those of PH, while melting and crystallization enthalpies increased by 10% (Supplementary Table S1). In some studies investigating the thermal properties of PCL/rGO composite materials, it was reported that T_c_, T_m_, especially $\chi_{c}$ (%) and melting enthalpy, increased with the addition of rGO, and that rGO had a positive effect on the thermal stability of the materials [31,53,54]. In our study, the impact of this material was overshadowed by the effect of HAp, since a small amount of rGO was used to avoid any potential toxic effects. However, the influence of rGO was observed especially in melting and crystallization enthalpies. 

### 3.8. TGA Analysis

TGA thermograms of P, PH and PHG are given in Figure 4c. The results indicated that the addition of HAp to the polymer lowered the temperature at 5% mass loss from 376.73 °C to 305.1 °C, whereas the temperature at the 50% mass loss decreased from 406.15 °C to 389.55 °C (Supplementary Table S2). This result can be explained by the fact that HAp causes a decrease in the thermal resistance of the polymer. In contrast, the addition of rGO to PH caused an increase in temperature (312.89 °C at 5% mass loss, and 397.1 °C at 50% mass loss). This could mean that rGO acts as a compatibilizer, increasing thermal stability. The results contradict some previous results in the literature of the decrease in decomposition temperature due to HAp content in the composition. This may be due to differences in material production method. Depending on its size and production method, HAp may cause an increase in thermal stability by creating extra molecular interactions with the polymer, or it may cause a decrease in thermal stability and the weakening of bonds by disrupting the homogeneity of the polymer. As demonstrated in many studies, rGO increases the thermal stability and degradation temperature of composite materials. In this study, the thermal stability of the polymer increased with the use of rGO. However, the thermal stability of PHG was still lower than that of P due to the integrated effect of HAp.

### 3.9. Compression Test

Compression test results of the constructs are given in Figure 4d,e. The maximum compression forces for the P, PH and PHG constructs were measured as 2643 N, 2717 N, and 3166 N, respectively. The maximum compressive stress values were also proportional to the compression force and were measured as 26.43 N/mm^2^, 27.17 N/mm^2^, and 31.66 N/mm^2^ in the same order. Considering that the compressive strength of the cancellous bone is in the range of 2–10 MPa (N/mm^2^), the values obtained for the constructs can be accepted to be at an appropriate level. As seen in Figure 4d,e, there was no significant increase in the compressive strength and stress of the micropatterned constructs after HAp addition; on the other hand the mechanical strength of the constructs containing HAp–rGO increased remarkably. Similarly, Seyedsalehi et al. (2020) reported a 50% increase in compression force and stress of rGO/PCL materials containing 0.5% rGO [49]; a decrease in compression force and tension was observed at rates of 1% and above.

### 3.10. Tensile Test

Figure 4f,g shows the tensile properties of P, PH, and PHG constructs. The maximum tensile force for P was measured as 29.66 N. Addition of HAp caused a ~38.74% reduction in maximum tensile strength; in addition, an insignificant decrease was observed after the addition of rGO. Similar results were obtained from the maximum tensile stress findings (Figure 4g). This can be explained by the stress points formed at the interface between polymer chains and particles (HAp and rGO), leading to a reduction in tensile strength, as noted in other studies [46].

### 3.11. In Vitro Hemocompatibility Test (ISO 10993-4)

**Figure 5:**
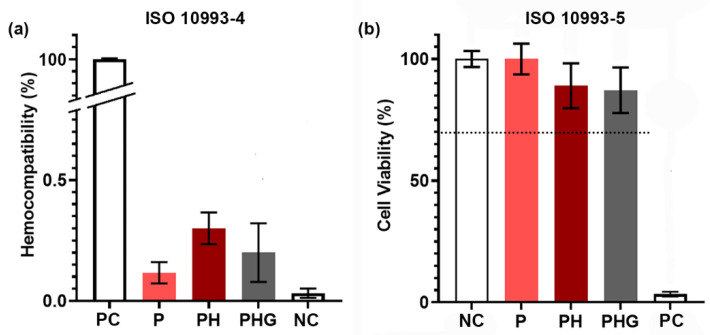
(**a**) In vitro hemocompatibility, and (**b**) indirect in vitro cytotoxicity of P, PH, PHG membranes (NC: negative control; PC: positive control). The dashed line at 70% in b indicates the lower limit of in vitro cytocompatibility.

Figure 5a demonstrates the in vitro hemocompatibility results of the P, PH and PHG. The percentages of hemolysis values of all the polymers are below 0.5% and all samples are hemocompatible according to the ISO 10993-4 [27]. The in vitro hemolysis test showed the hemocompatibility of P, PH, PHG. As a result of the inclusion of HAp in the composition, the blood hemolysis value increased slightly but insignificantly. This may be explained by the increase in the pH of the environment due to the alkaline release from HAp [42]. The addition of rGO made polymers more compatible by decreasing their hemolysis values slightly.

### 3.12. Indirect In Vitro Cytotoxicity Test (ISO 10993-5)

In vitro cytotoxicity analysis was performed to determine if there was any toxicity as a result of the material content or the manufacturing procedure. The results are presented in Figure 5b. As it is known, rGO can show cytotoxicity at high concentrations [55]. Therefore, it is essential to exploit the contribution of rGO to the biomaterial content without creating a toxic effect on hOBs. The analysis results showed that the cell viability values determined for P, PH and PHG were 99.98%, 88.99%, and 87.15%, respectively, and all groups could be defined as cytocompatible according to ISO10993-5.

### 3.13. SEM Analysis of Cell Laden Contructs

**Figure 6:**
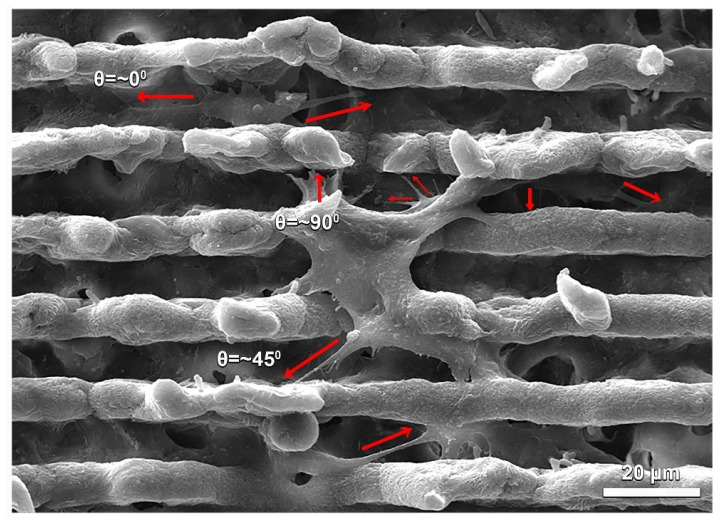
Representative SEM image demonstrating the orientation of human osteoblast cells with respect to groove direction on the first day of culture on a PH-2 membrane. Similar cell orientation was observed on different materials (P, PH, PHG) and pattern types (1 and 2) (Supplementary Figure S2).

Figure 6 shows a representative SEM image of hOB morphology on the initial (1st) day of culture on PH membrane. Regardless of the membrane content and pattern dimensions, the cells spread between the channels or adhered to ridges of the pattern and they laid down in the same direction as the pattern orientation. Figure S2 shows the substantially similar orientation behavior of hOBs on different materials (P, PH, PHG) and pattern types (one and two). Many studies have reported that micro-patterns, in particular, support cell attachment [21,22,23]. This feature is important for cells to form colonies in prospective time periods. The cells attach to the ridges of the pattern with multiple pseudopodia. These pseudopods form an orientation angle with the direction of the grooves [22]. The orientation angles of the cells with respect to groove direction are between 0° and 90° as shown in Figure 6. When the average orientation angle is around 45° or higher, cells spread in the lateral direction with respect to the groove. In the case that the orientation angle is 10° or less, cells usually spread in the same direction as the groove.

**Figure 7:**
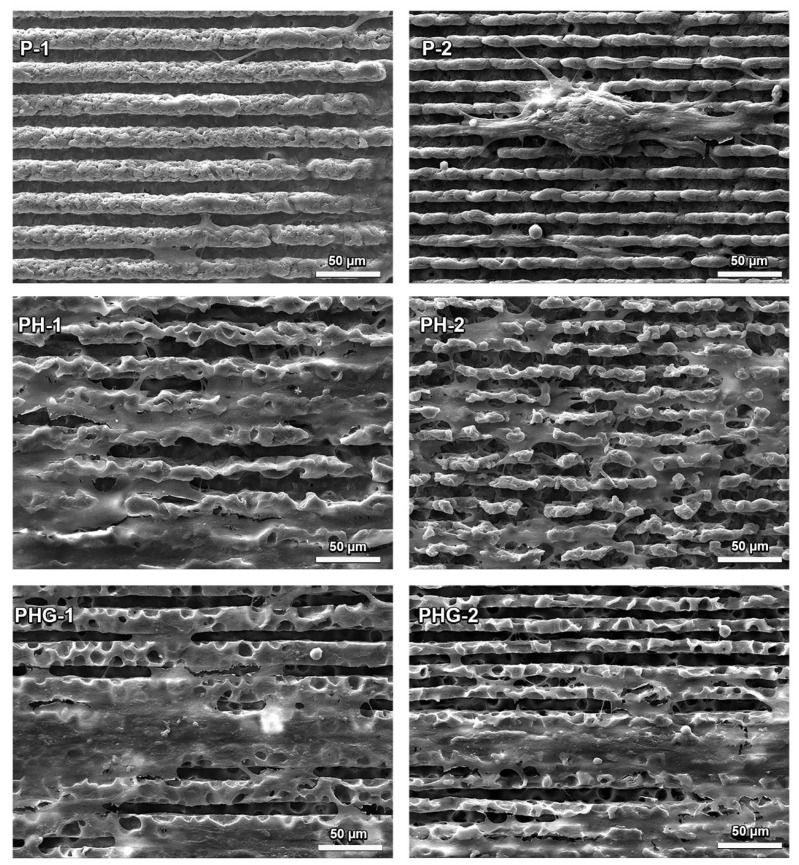
SEM images of human osteoblast cells cultured for 7 days on membranes of P, PH, PHG membranes with two different pattern sizes.

SEM images of P, PH, PHG membranes cultured with hOBs on the seventh day of culture are given in Figure 7. It is clearly seen from the SEM images that P had the lowest cell density compared to other groups. On day 7, PH and PHG membranes were almost completely covered with cells (Figure 7).

The groove structure also affected the morphological properties of the cells. Normally, osteoblasts displaying a rounded morphology got stuck between the channels in an elliptical form. The shape of the cell is also important as the initial attachment behavior, as it affects the growth and life span of the cell, as well as many metabolic activities [55].

The pattern determined the initial attachment and cytoplasm morphology as well as the migration direction of the cell colony. In particular, the filopodia extending in the same direction as the pattern was considered as an indication of this [56]. As the cell colonies overlapped in the later days of the culture, the pattern was partly covered with cells; hence, the surface pattern could not be distinguished with SEM. However, even in this case, it has been observed that the cells lie down on the same orientation as the pattern, consistent with the observation of Papenburg et al. (2007) [18].

### 3.14. MTT Analysis

**Figure 8:**
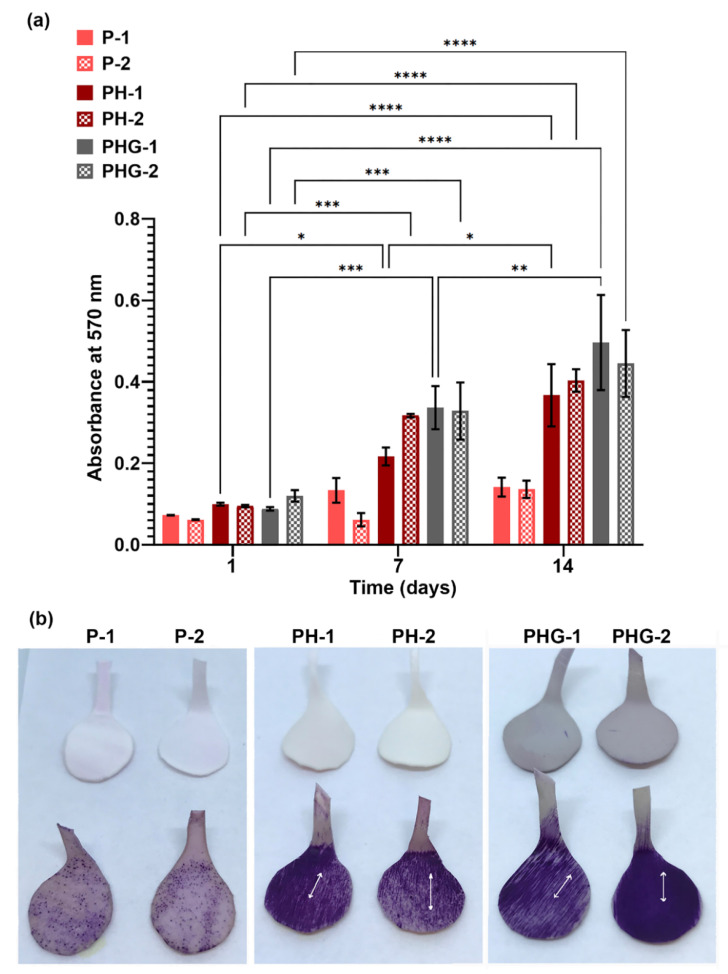
(**a**) In vitro viability of osteoblast cells cultured on P, PH, PHG membranes for up to 14 days based on MTT assay. (**b**) Macroscopic images demonstrating the formation of formazan crystals on the P, PH and PHG constructs at 14th day of culture. Arrows indicate the direction of the grooves on the patterns. Circular-cut membranes are 9.8 mm in diameter; tails were left for ease of handling. Statistical significance; * *p* ≤ 0.05, ** *p* ≤ 0.01, *** *p* ≤ 0.001, **** *p* ≤ 0.0001.

hOBs viability on P, PH and PHG constructs was also analyzed by MTT assay at the 1st, 7th and 14th days of culture, and the results are shown in Figure 8. On the first day of culture, there was no significant difference in cell viability depending on the content or surface pattern of constructs. On the seventh day of culture, the cell density on P-1 increased nearly by 84% compared to that of the first day of culture. From the 7th day to 14th day, the cell density on P-1 increased slightly by 6%. On the other hand, the cell density on P-2 remained constant at the first day and seventh day. On the 14th day of culture, the cell densities on P-1 and P-2 reached the same value. The results indicated that incorporation of HAp enhanced cell proliferation. On the seventh day, the cell density was found to be more than two-fold of that on the first day. On the 14th day, the cell density increased approximately by 70% compared to that of the 7th day. The low concentration of rGO used for the preparation of PHG influenced cell proliferation significantly. The cell density on PHG increased threefold from the first day to the seventh day. The absorbance value of cells cultured on PHG was increased ~45% on the 14th day compared to that of the 7th day. In three constructs, the effect of pattern type on cell viability was not observed on the first day of culture. On the seventh day, the cell density depending on pattern type began to be apparent. For example, the cell density on P-1 was found to be higher than that on P-2. Similarly, the cell density on PHG-1 was found to be higher than that on PHG-2, whereas cell density on PH-2 was higher than that of PH-1. However, on the 14th day, the differences in viability arising from pattern types disappeared and the cell densities on different patterns of the same constructs became nearly the same (Figure 8a).

Figure 8b shows the formazan crystals formed on constructs at the 14th day of culture. The macroscale images are fully consistent with the absorbance values. As seen in the images, the density of the formed formazan crystals on PH and PHG was higher than that of P. The highest amount of formazan crystal formation was observed in the PHG group. Normally, the patterns on the membrane surface are of micron size and cannot be distinguished by the naked eye. However, the macroscale images showed that the cells proliferated on the constructs in the same direction with the pattern orientation, and the purple formazan crystals formed a pattern in the same direction as the pattern.

### 3.15. Time-Based alamarBlue Cell Viability Analysis

**Figure 9:**
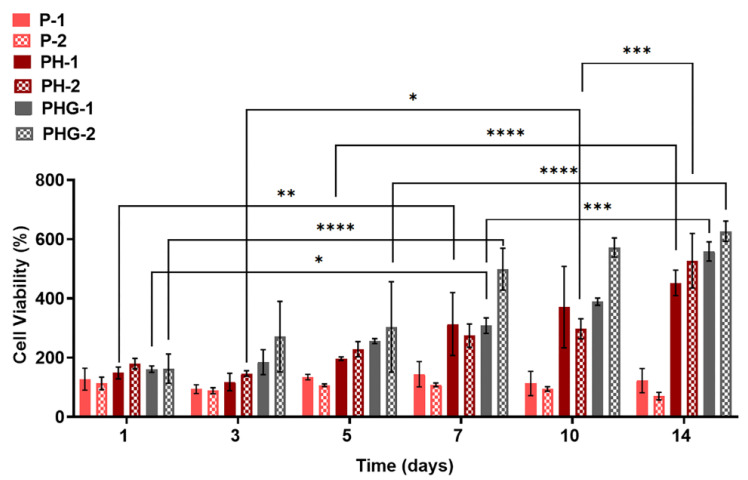
Metabolic activity of osteoblast cells cultured on P, PH, PHG membranes for up to 14 days based on alamarBlue assay. Statistical significance; * *p* ≤ 0.05, ** *p* ≤ 0.01, *** *p* ≤ 0.001, **** *p* ≤ 0.0001.

Figure 9 shows the percentages of cell viability on 1st, 3rd, 5th, 7th, 10th and 14th day of culture obtained from the alamarBlue assay. The results indicated that the viability of hOBs cultured on P did not change significantly over 14 days. Although P is a suitable material for cell viability and cell attachment (and can be considered as positive controls for PH and PHG groups), it was not sufficient to support cell proliferation. The viability of hOBs cultured on PH was not changed significantly during the first three days. The cell viability began to increase gradually from the fifth day of culture. The cell viability on the 14th day was found to be 2.5-fold that of the 1st day. 

The viability of hOBs cultured on PHG increased more than 50% at day 3 than that of day 1. On the 10th day of culture, the cell viability increased almost 100% compared to day 1. On the fourteenth day, the cell viability was found to be threefold higher than that on the first day. There were no significant differences in the cell viability on P and PH constructs caused by different forms of patterns. However, the percentages of viability of cells on PHG-2 were found to be higher than that of PHG-1 on the 7th and 10th day of culture. At the 14th day of culture, the percentages of viability on different patterns of PHG had reached approximately the same values.

### 3.16. Osteogenic Activity of Cells on Membranes

**Figure 10:**
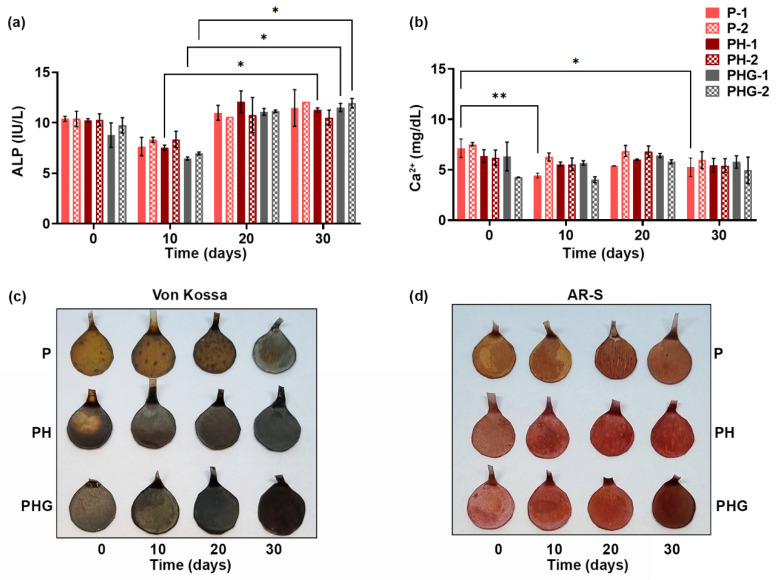
Osteogenic activity of cells cultured on P, PH, PHG membranes: (**a**) Alkaline phosphatase activity and (**b**) extracellular calcium secretion of the osteoblasts up to 30 days. Macrographs showing histochemical stainings: (**c**) von Kossa, and (**d**) Alizarin red S-stained samples. Circular cut membranes are 9.8 mm in diameter; tails are left for ease of handling. Statistical significance; * *p* ≤ 0.05, ** *p* ≤ 0.01.

ALP activity and extracellular calcium concentrations of hOBs are shown in Figure 10a,b, respectively. The cells cultured on the micropatterned membranes maintained ALP activity throughout the 30 day culture period. ALP activity increased gradually from 10th day to 30th day of culture (Figure 10a). On the other hand, the concentration of extracellular Ca^2+^ of the cells on PH and PHG membranes remained nearly constant during culture (Figure 10b). Apart from that, no significant difference could be detected in the ALP activity or Ca^2+^ secretion of cells depending on content and pattern of the constructs they had attached. It should be kept in mind that these cells are not stem cells (e.g., mesenchymal stem cells) that are differentiating into an osteogenic lineage on the membranes. It is important that the hOBs maintained their existing osteogenic activity on the membranes over the fairly long 30-day culture period.

Findings of the histochemical stainings of cell-laden constructs are presented in Figure 10c,d. Although the PH and PHG constructs can be stained to some extent due to their HAp content (day 0 staining), the increase in staining intensity of calcium deposits (in black/brown color for von Kossa, and in orange/red color for Alizarin red S) over time can be considered as an indicator of cell-mediated mineralization on the constructs. Considering both von Kossa and Alizarin red S stainings, it was observed that the staining intensity was in order of PHG > PH > P. Secondly, it was observed that the staining intensities increased gradually during the culture time. Taken collectively, these findings reveal that PH and PHG structures in particular are suitable substrates for the mineralization of osteogenic cells.

## 4. Conclusions

In this study, the PSµM technique was applied to develop micro-patterned ternary blend membrane constructs made up of poly(ε-caprolactone), nano hydroxyapatite and reduced graphene oxide. Symmetric and repetitive micro groove channels enhanced adhesion and directional mobility and also induced the elliptical structure of osteoblasts. In the case of specific guidance of the cell proliferation, such a micropatterned surface may provide an appealing approach. HAp and rGO promoted cell proliferation as a bioactive scaffold. Although the amount of rGO in the composition had a minor effect on the physical and chemical properties of the membranes, the effect on cell proliferation was noticeable. alamarBlue and MTT tests indicated that the membranes, especially PH and PHG, support cell viability even when cell colonies grew in overlayers. SEM images showed that despite overgrown cell colonies, pattern orientation was still preserved. Micro-grooved constructs developed in membrane form may have potential use in orthopedic or maxillofacial surgeries, such as for the reconstruction of periosteal tissue removed because of osteosarcoma or osteomyelitis, or for use as a neo-periosteal flap in articular chondrocyte implantation procedures using the periosteum cover technique. In addition, these constructs may also have potential for in vitro applications such as cell biosensors, microfluidic chips or tissue models. However, well-designed prospective in vitro and in vivo studies will be able to show the suitability of the developed constructs for potential application areas.

## Figures and Tables

**Table 1:** Pattern dimensions (in µm) of silicon wafers.

Pattern Code	C (Channel)	Ridge (R)	Depth (D)
1	20	10	20
2	10	10	20

**Table 2:** Expected pattern dimensions (in µm) of the constructs.

Pattern Code	C (Channel)	Ridge (R)	Height (H)
1	10	20	20
2	10	10	20

**Table 3:** Chemical compositions and codes of the patterned constructs for the solvent (10 mL 1,4-dioxane) and non-solvent (200 mL EtOH) pair.

Construct Code	PCL	HAp	rGO
P	1.82 g	-	-
PH	1.82 g	0.364 g	-
PHG	1.82 g	0.364 g	1 mg

**Table 4:** Measured pattern sizes (in μm) for P, PH and PHG constructs.

Constructs	Pattern 1		Pattern 2	
	Channel	Ridge	Channel	Ridge
P	10.01 ± 0.45	17.24 ± 0.68	9.04 ± 0.81	8.83 ± 0.62
PH	9.47 ± 0.94	17.87 ± 0.86	8.33 ± 1.03	9.04 ± 0.64
PHG	9.12 ± 0.95	17.43 ± 0.81	8.80 ± 0.87	8.38 ± 0.78

## Data Availability

The data that support the findings of this study are available from the corresponding author upon reasonable request.

## Supplementary Materials

The following supporting information can be downloaded at: https://www.mdpi.com/article/10.3390/molecules27207091/s1, Figure S1: SEM micrographs of P, PH, PHG membranes fabricated by two different pattern dimensions; Figure S2. SEM micrographs demonstrating the orientation of human osteoblast cells with respect to groove direction on the first day of culture on P, PH, PHG membranes with different pattern types (1 and 2); Table S1: Crystallization and melting temperatures and melting enthalpies calculated based on DSC cooling and secondary heating analysis; Table S2: Temperature (in °C) values and ash residue amounts corresponding to 5% and 50% mass losses as a result of thermogram analysis.
