# Supplementary material for: Evaluation of Human Osteoblasts on NIPS Micro-Patterned PCL Carriers Containing Nanohydroxyapatite and Reduced Graphene Oxide Using PSµM
Source: Molecules. 2022 Oct 20;27(20):7091. doi: 10.3390/molecules27207091 (PMC9609095; doi:10.3390/molecules27207091)
Supplement: Supplementary file 1 [file molecules-27-07091-s001.zip › molecules-1899491-supplementary.pdf]

Supplementary Materials

Evaluation of Human Osteoblasts on NIPS Micro-Patterned PCL Carriers Containing Nanohydroxyapatite and Reduced Graphene Oxide Using PSμM

Burcu Tüzün-Antepli [1], Şükran Şeker [1], Ayşe Eser Elçin [1], Gilson Khang [2] and Yaşar Murat Elçin [1,3,*]

[1] Tissue Engineering, Biomaterials and Nanobiotechnology Laboratory, Ankara University Faculty of Science, Ankara University Stem Cell Institute, 06100 Ankara, Turkey
[2] Department of BIN Convergence Technology, Department of Polymer Nano Science and Technology, Jeonbuk National University, Jeonju 54896, Jeonbuk, Republic of Korea
[3] Biovalda Health Technologies, Inc., 06830 Ankara, Turkey

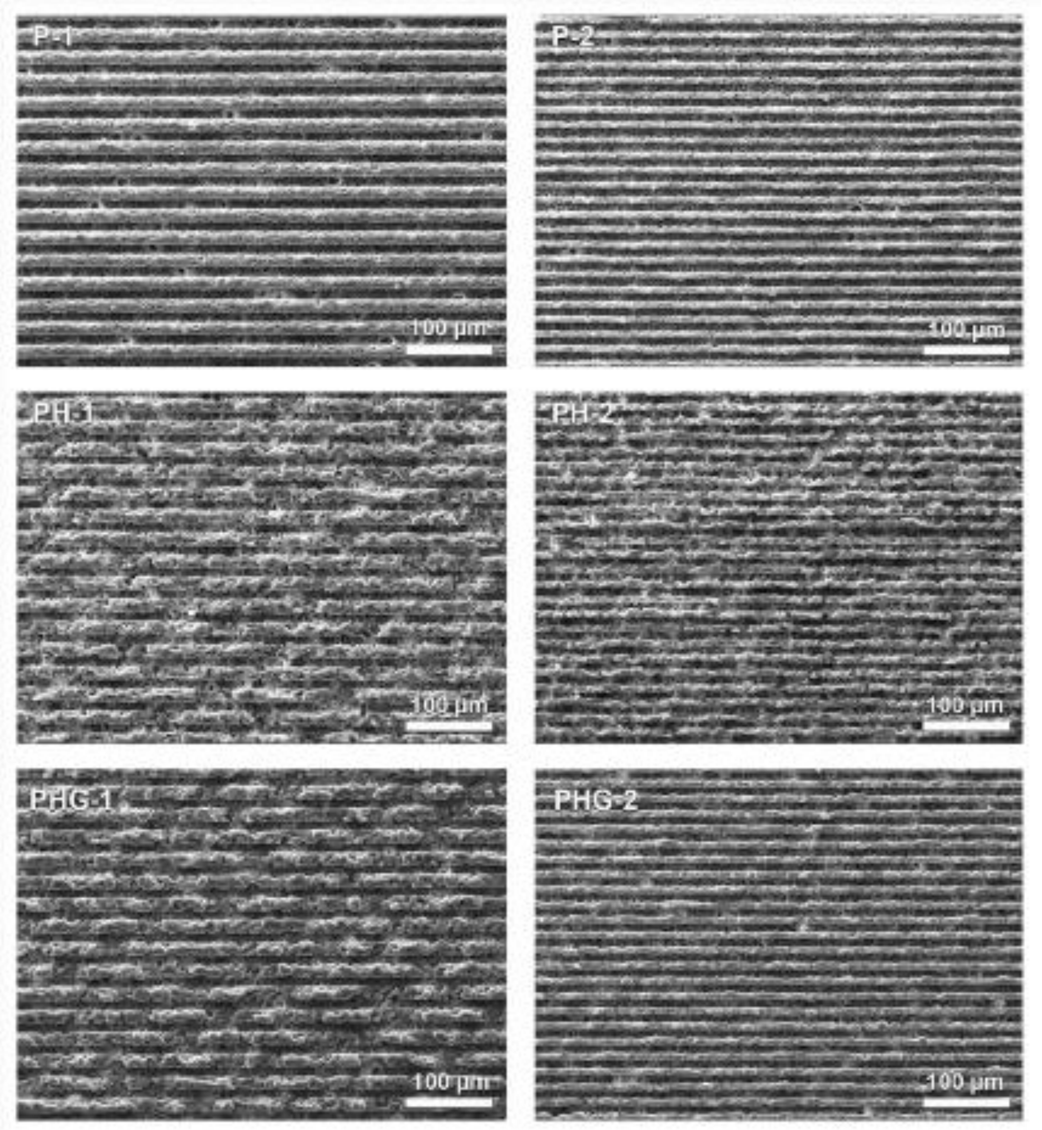

Figure S1. SEM micrographs of P, PH, PHG membranes fabricated by two different pattern dimensions. Silicon wafer pattern dimensions are; C: 20 μm, R: 10 μm, D: 20 μm for pattern #1, and C: 10 μm, R: 10 μm, D: 20 μm for pattern #2.

Table S1. Crystallization and melting temperatures and melting enthalpies calculated based on DSC cooling and secondary heating analysis

	P	PH	PHG
T_c (°C)	30.51	33.28	33.34
ΔH_c (J/g)	-54.422	-44.279	-49.815
T_m (°C)	56.22	57.91	56.91
ΔH_m (J/g)	57.431	44.085	49.999
χ_c (%)	41.1692	37.9225	43.02958565

.

Table S2. Temperature (in oC) values and ash residue amounts corresponding to 5% and 50% mass losses as a result of thermogram analysis.

Constructs	T (5%)	T (50%)	Residual (%)
P	376.73	406.15	2
PH	305.1	389.55	20
PHG	312.89	397.1	17

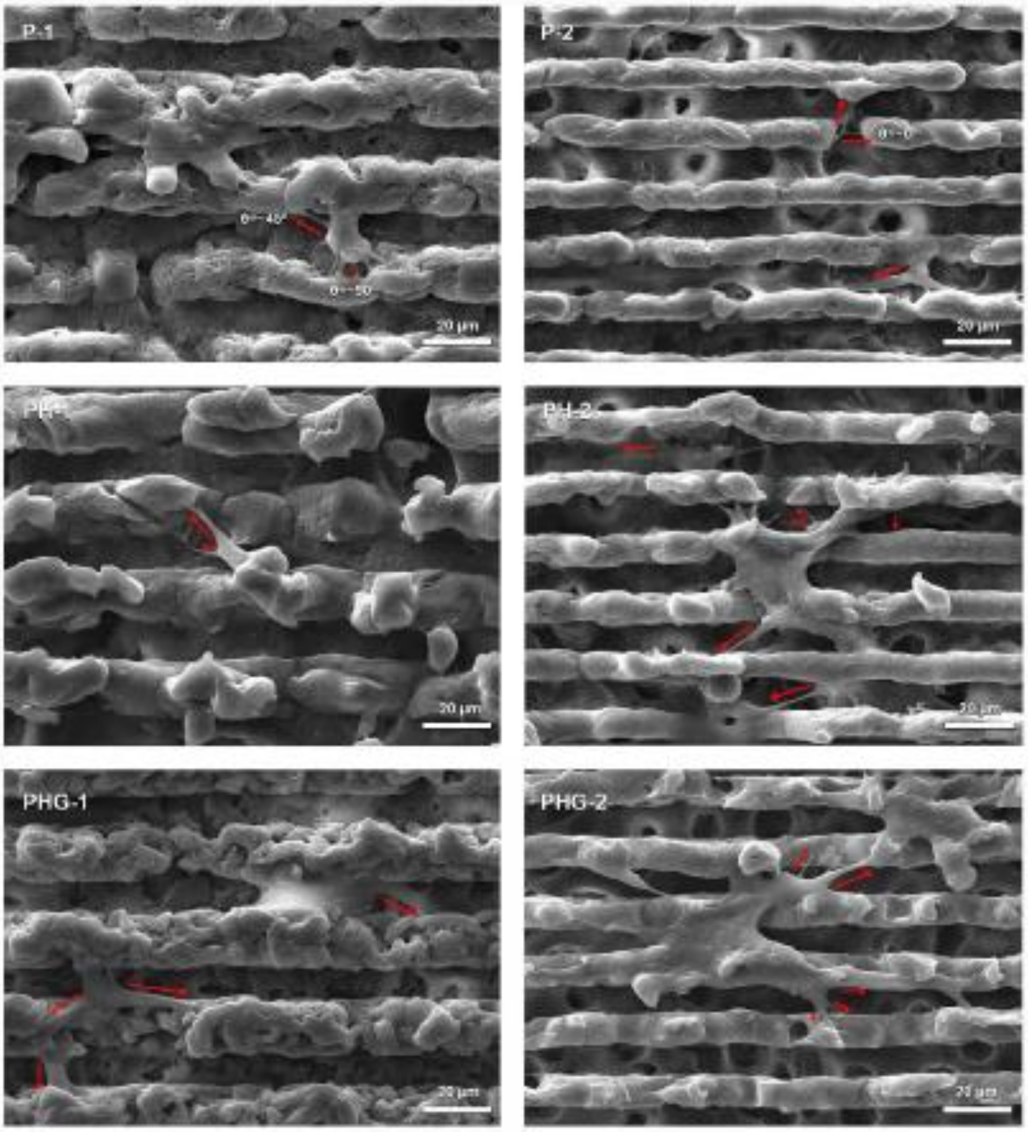

Figure S2. SEM micrographs demonstrating the orientation of human osteoblast cells with respect to groove direction on the first day of culture on P, PH, PHG membranes with different pattern types (1 and 2). Although the cell proliferation levels may differ, similar cell orientations were observed in different membrane types. The orientation angles of the cells with respect to groove direction are between 0o and 90o. When the average orientation angle is around 45o or higher, cells spread lateral direction with respect to groove. .
